# The Future of Minimal-Access Myoma Surgery with In-Bag Contained Morcellation

**DOI:** 10.3390/jcm12113628

**Published:** 2023-05-23

**Authors:** Rajesh Devassy, Rohan Rajesh Devassy, Maya Sophie de Wilde, Harald Krentel, Aizura Adlan, Luz Angela Torres-de la Roche, Rudy Leon De Wilde

**Affiliations:** 1 Department of Obstetrics and Gynecology, Minimal-Access Surgery & Oncology, Dubai London Clinic and Speciality Hospital, Dubai 3371500, United Arab Emirates; 2 Faculty of Medicine, Kasturba Medical College, MAHE, Mangalore 575001, Karnataka, India; 3 University Hospital for Gynecology, Pius Hospital, University Medicine Oldenburg, 26121 Oldenburg, Germany; 4 Clinic of Gynecology, Obstetrics, Oncology and Senology, Bethesda Hospital, 47053 Duisburg, Germany; 5 Department of Obstetrics and Gynaecology, Faculty of Medicine, University of Malaya, Kuala Lumpur 50603, Malaysia; aizura@um.edu.my

**Keywords:** morcellation, myomatous tissue, contained closed bags, specimen extraction, leiomyoma, laparoscopy

## Abstract

Contained electromechanical morcellation has emerged as a safety approach for laparoscopic myomatous tissue retrieval. This retrospective single-center analysis evaluated the bag deployment practicability and safety of electromechanical in-bag morcellation when used for big surgical benign specimens. The main age of patients was 39.3 years (range 21 to 71); 804 myomectomies, 242 supracervical hysterectomies, 73 total hysterectomies, and 1 retroperitoneal tumor extirpation were performed. A total of 78.7% of specimens weighed more than 250 g (n = 881) and 9% more than 1000 g. The largest specimens, weighing 2933 g, 3183 g, and 4780 g, required two bags for complete morcellation. Neither difficulties nor complications related to bag manipulation were recorded. Small bag puncture was detected in two cases, but peritoneal washing cytology was free of debris. One retroperitoneal angioleiomyomatosis and three malignancies were detected in histology (leiomyosarcoma = 2; sarcoma = 1); therefore, patients underwent radical surgery. All patients were disease-free at 3 years follow-up, but one patient presented multiple abdominal metastases of the leiomyosarcoma in the third year; she refused subsequent surgery and was lost from follow-up. This large series demonstrates that laparoscopic bag morcellation is a safe and comfortable method to remove large and giant uterine tumors. Bag manipulation takes only a few minutes, and perforations rarely occur and are easy to detect intraoperatively. This technique did not result in the spread of debris during myoma surgery, potentially avoiding the additional risk of parasitic fibroma or peritoneal sarcoma.

## 1. Introduction

For almost 30 years, endoscopic surgery has been positioned as the standard approach for the management of gynecological tumors in many countries [1,2,3,4,5], despite the technical considerations related to tumor manipulation and retrieval [6,7,8,9,10,11]. In 1995, the USA FDA approved electromechanical morcellation to retrieve surgical specimens during endoscopic surgery, but after decades of experience with this technique, in 2014, the FDA warned about the potential risk of postsurgical parasitic myoma development and, especially, occult sarcoma dissemination secondary to tissue spieling during myomata and uteri morcellation [6,12,13]. Although the incidence of uterine sarcoma is estimated to be low or very low at 0.06%, the dissemination of the disease in women undergoing hysterectomy for benign indications has been proven to be associated with uterine tissue morcellation [1,14,15,16]. It is clear that these risks should be avoided and balanced with the benefits of modern minimally invasive techniques [17,18], such as less postsurgical pain, fewer complications, less in-hospital stay, and a rapid return to daily activities, when compared with laparotomy [1,12]. The challenge for physicians is to find a scientifically validated solution without moving to traditional open surgery and to avoid future medico-legal problems related to postsurgical parasitic myoma development and occult sarcoma dissemination [19]. For this purpose, in-bag contained morcellation (IBM) systems were developed to facilitate a safe surgical specimen retrieval and were eventually approved for human use by the medical regulatory agencies in 2020 [20].

Due to their recent introduction, it is necessary to evaluate the feasibility, safety, and efficacy of these systems to avoid surgical tissue spreading, as well as to set out guidelines for proper patient and morcellation technique selection [1]. Therefore, in this study, we evaluated the bag deployment practicability and the safety of electromechanical in-bag morcellation (IBM) to prevent tissue spillage when used to retrieve big surgical specimens.

## 2. Materials and Methods

Retrospective single-center analysis of 1120 patients who underwent laparoscopic uterine surgery and electromechanical IBM for presumed benign pathology, between 2 July 2014 and 14 December 2021, at the Department of Obstetrics and Gynecology, Minimal-Access Surgery and Oncology, Dubai London Clinic and Speciality Hospital, Dubai, UAE. Criteria for inclusion were consented cases in which laparoscopic myomectomy or hysterectomy with in-bag morcellation was performed for presumably benign myomatous pathology. Cases with uterine diseases other than LM or treated outside the proposed time range were excluded. Here we present the results related to type of surgery, bag deployment practicability, intraoperative complications, bag puncture, final peritoneal lavage cytological analysis, and specimen weight and histology. Regarding bag deployment practicability, the following steps during bag handling were described: (a) bag introduction, (b) bag straightening, (c) achieving clear vision, (d) appropriate insufflation and adherence to the port size and sealing, (e) specimen introduction, (f) morcellation, (g) extraction of blood, smaller tissue or fluid debris, and (h) bag extraction.

For the analysis, descriptive statistics were used. The bags consist of thermoplastic polyurethane, which is a medical-grade polymer widely used in the medical industry (MorSafe, Veol medical technologies PVT LTD, Mumbai, India) [12], and are available in different sizes that could contain up to 5500 mL of morcellated tissue (Table 1). The retrospective nature of this study did not require the approval of an ethical committee; only IRB permission was obtained for this analysis.

## 3. Results

A total of 1120 patients underwent laparoscopic uterine surgery and electromechanical IBM for presumed benign pathology. The average age of the patients was 39.3 years, ranging between 21 and 71 years, with weight from 41.0 Kg to 127.6 Kg (median 65.5 Kg). In addition, 50.2% were nulliparous, and 71.78% of all cases underwent myomectomy. In total, 71.24% of surgical specimens weighed between 250 g and 999 g, and 7.23% were larger than 1000 g (Table 2).

It was found that, prior to the operation, all patients signed an informed consent for the use of the electromechanical morcellation system MorSafe (Veol Medical Technologies Pvt. Ltd., Mumbai, India) as well for the use of surgical bags made of thermoplastic polyurethane. In accordance with their presurgical diagnosis and patient desire, patients underwent hysterectomy or myomectomy. Perioperative intravenous antibiotic prophylaxis (Lomefloxacin, 1 g) was used in all cases.

**Figure 1:**
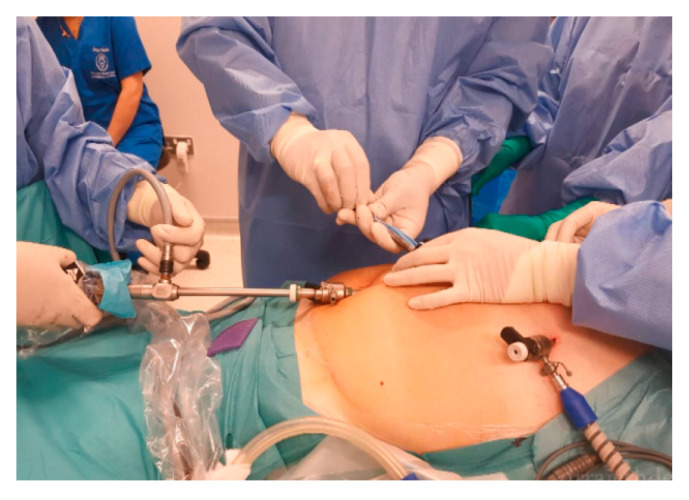
Trocar placement configuration to access big abdominal tumors.

Regarding the technique used for a big specimen, Figure 1 shows how the trocars were placed to manipulate tumors and bags. After assessing the size and number of the myoma, a typical laparoscopic myomectomy began by placing the telescope port 3–4 finger widths from the highest point of the uterus or fibroid fundus. After the inspection of the abdominal cavity, ancillary ports were placed with a minimum of two 5–6 mm trocars and one 10–12 mm trocar. The size of the tertiary trocar depends upon the size of the myoma. In myomas measuring less than 10 cm, a 5–6 mm trocar is preferable, while for those above 10 cm or multiple myomas, a 10–12 mm trocar is opted for as tertiary trocar.

**Figure 2:**
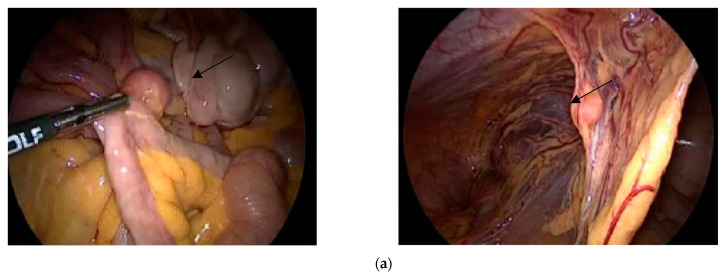

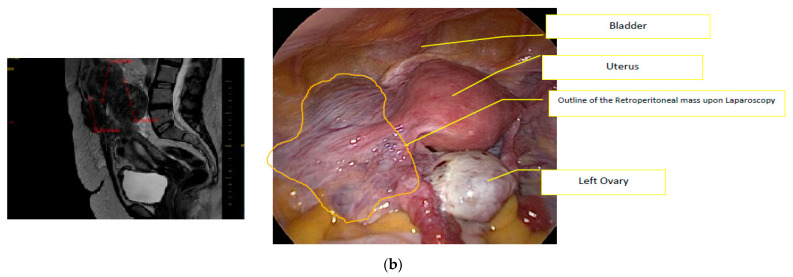
Giant retroperitoneal angioleiomyomatosis tumor as seen at MRI and at initial diagnostic laparoscopy. (**a**) Arrows show parasitic myomata between intestines (**left**) and within a peritoneal adhesion (**right**). (**b**) Giant retroperitoneal angioleiomyomatosis tumor as seen at MRI and at initial diagnostic laparoscopy.

Ten cases of parasitic myomata were found in patients who had undergone previously uncontained electronic morcellation during laparoscopic myomectomy or hysterectomy (Figure 2a). One retroperitoneal angioleiomyomatosis tumor (Figure 2b) was found in a patient who was clinically diagnosed as a giant ovarian neoplasm. At the preoperative image examinations (ultrasound, MRT, and PET-CT), this mass was described as a large multilobulated left pelvic soft mass lesion (6.6 cm AP × 5.1 cm T × 7.7 cm CC).

Histological examination revealed the presence of fibroids in most of the cases (78%) (Table 3). This was consistent with the preoperative evaluation of all patients by ultrasound and contrast-enhanced MRI scan with diffusion-weighted imaging (DWI) and calculation of apparent diffusion coefficient (ADC) values. On radiologic examination, leiomyosarcoma was not suspected, and patients did not have elevated LDH levels. However, intraoperatively, two cases of leiomyosarcoma and one endometrial stromal tumor were diagnosed by frozen biopsy, which were suspected due to the great fragility of the tissue when grasped, even when manipulated with blunt forceps.

The first case was a 33-year-old nulliparous woman who presented with severe menorrhagia and secondary anemia because of a degenerated 6–7 cm myoma (FIGO type 1–2). She had given a history of a myomectomy in 2011, performed by laparotomy in another country; thus, the details and definitive histology of the resected myoma were preoperatively unknown. For the new clinical situation, the patient underwent laparoscopic myomectomy and in-bag extraction through the umbilicus. Due to high tissue fragility, probably because of the degenerative nature of the myoma, mechanical morcellation was not necessary. Frozen biopsy and definitive histology reported an atypical myoma with bizarre nuclei. One year later, the patient complained of pelvic pain. At this time, the patient brought a copy of the surgical and histological report of the first myomectomy that showed leiomyosarcoma. However, she had not received instructions on close follow-up. The new ultrasonographic and pelvic MRI examination revealed a large mass in the left adnexal area with solid and cystic components. The PET showed no evidence of metastatic disease. Therefore, in accordance with our tumor board recommendations, a total abdominal hysterectomy, left salpingo-oophorectomy, and right and left iliac obturator lymphadenectomy were performed. The 259 g tumor was reported as uterine leiomyosarcoma with negative margins, stage 1b (TIbNOMO). The patient moved to London and continued her treatment there. A few years later, she returned to Dubai and presented with a relapse with multiperitoneal lesions, for which she was referred to a cancer center to continue her treatment.

The second case was a 46-year-old nulliparous woman who presented with a large fundal myoma, severe secondary dysmenorrhea, and anemia. Preoperative imaging tests and endometrial biopsy were negative for malignancy. The patient underwent in-bag laparoscopic myomectomy, and due to tissue fragility, the manually morcellated surgical specimen was removed through a suprapubic minilaparotomy. A total of 3183 g of myoma tissue was obtained and reported as leiomyosarcoma. The patient did not consent to the recommended radical hysterectomy and lymphadenectomy and was lost to follow-up.

The third case was a 58-year-old parous woman with a history of pelvic pain and postmenopausal bleeding due to myomatosis uteri. No signs of malignancy were present at the preoperative ultrasound examination, PAP cytology, and endometrial biopsy. During the LASH, the frozen biopsy revealed a grade 1 endometrial stromal adenocarcinoma with >50% invasion of myometrium; the tubes and ovaries were reported normal. In addition, enlarged para-aortic lymph nodes were found. Consequently, a radical hysterectomy, bilateral salpingo-oophorectomy, partial vaginectomy, para-aortal lymphadenectomy, and bilateral parametria excision were performed. Postoperatively, six cycles of adjuvant chemotherapy with paclitaxel and carboplatin were given. Follow-up examinations showed the absence of cancer recurrence over 5 years.

Regarding the angioleyomiomatosis case, sections of the soft tissue consisted of cellular sheets and irregular fascicles spindled cells with the formation of glomeruloid architecture and epithelioid features. The lesion demonstrated numerous anastomosing structures resembling blood vessels covered by endothelium-like cells embedded in fibrous stroma. There was no evidence of malignancy or tumor necrosis. The tumor was positive for CD31, CD34, D2-40, CD68, Ki-67 (1% to 2% positive nuclear staining) immunomarkers as well for the smooth muscle markers SMA and desmine. The markers HHV-8, S100, synaptophysin, and calretinin were negative. The cytology of peritoneal fluid showed mild subacute inflammation with activated mesothelial cells suggestive of exudative peritoneal effusion [21]. The peritoneal washing cytology examination of all specimens did not show any case of malignancy or tissue spillage (Table 4).

Regarding the assessment of the “practicability of in-bag morcellation”, the bags were selected according to the volume of the specimen size in all cases. The mean intraoperative time necessary for the introduction of the endobag was 0:16:52 min, and the mean morcellation time 0:22:51 min (Table 5). Only in five cases (0.45%) was extensive manipulation observed due to multiple fibroids or due to the large size and diameter of the specimen, which made it difficult to introduce it into the bag.

**Figure 3:**
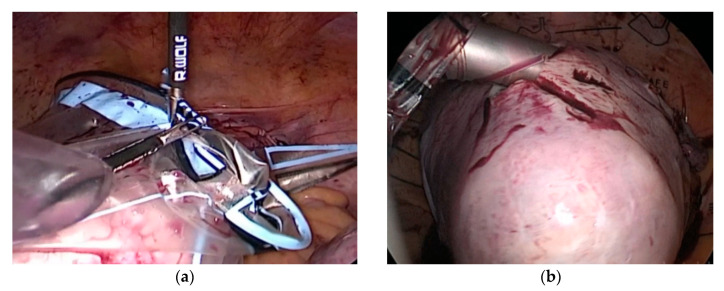
Visibility of a medical thermoplastic polyurethane surgical bag during morcellation of a giant myoma (2800 g): (**a**) closing the large bag; (**b**) initial morcellation.

The large bags enabled the trouble-free morcellation of big species up to 2800 g (Figure 3). Transfer of the X-L bag into the abdomen was more time-consuming—almost double—than other sizes of bags, which was normally less than 1 min, mainly due to the longer time required for bag deployment. The introduction of larger masses in the smaller abdomen was the most difficult step. There were no cases of bag handling failure, nor were there any cases of failed morcellation.

**Figure 4:**
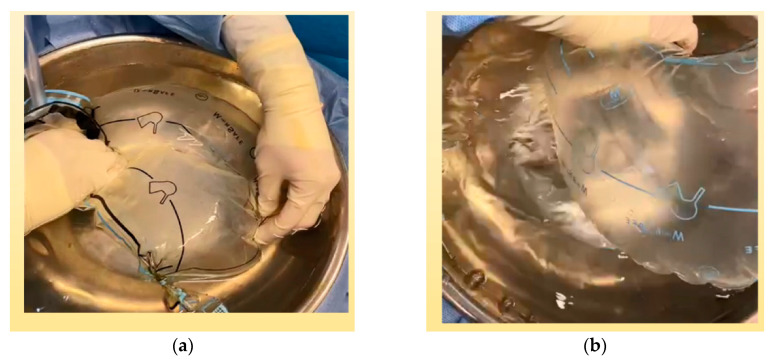
Postmorcellation insufflation and immersion test of medical thermoplastic polyurethane surgical bags: (**a**) negative test; (**b**) positive test showing burbles in water.

Two bag punctures occurred. One case was by direct contact with the morcellator blade at the edge of the bag, near the port outlet, which allowed surgeons to exteriorize the section immediately, avoiding any spillage. The other was a microrupture that did not lead to a loss of gas from the bag and was adverted postoperatively at the insufflation and immersion test. This test was routine at the end of each procedure performed (Figure 4). There were no intraoperative or postoperative bag-induced complications such as injury, bleeding, or infections.

All patients, including those with benign and rare cellular types, had regular annual follow-ups, the longest period being 8 years. No myoma recurrence was observed. One patient with leiomyosarcoma was free of disease at the 5-year follow-up, and the other did not present again at follow-up. There were no hernias at the port sites.

## 4. Discussion

Leiomyomata are monoclonal benign tumors that arise from myometrial cells with complex pathobiological origins involving genetic, epigenetic, hormonal, environmental, proinflammatory, angiogenetic, and growth factors [22]. Uterine myomata may present as single or multiple tumors easily recognized through ultrasound examination, although MRI or CT are sometimes required for further therapeutic decision-making. The associated symptoms depend on the size and location of the tumor; fibroids greater than 4 cm tend to hamper fertility and pregnancy outcomes or lead to chronic anemia or pelvic pain [22]. Besides myomectomy and hysterectomy (via laparotomy or endoscopic approach), a broad spectrum of options is available for the treatment of myomas, including pharmacological myoma growth control (GnRH analog, ulipristal) and nonsurgical procedures (artery embolization, radiofrequency ablation, sonography-guided transcervical fibroid ablation, high-intensity focused ultrasound ablation). The election should be based on risk–benefit analysis, depending on the patient’s desire, size, number, and location of the fibroids and the requirement of a multistage approach to obtain a reduction in the fibroid or its related symptoms [23]. The endoscopic management of large and giant uterine myomata, which are not frequently described [24], requires the use of morcellation for surgical specimen retrieval.

At our clinic, prior to 2014, morcellation was always performed without a bag, and here we presented the retrospective analysis of 1120 cases of big and giant uterine myomatosis performed between 2014 and 2021 to describe the feasibility and safety of laparoscopic contained morcellation for the retrieval of large surgical specimens from the abdominal cavity. According to our experience, advancements in minimally invasive surgical techniques and tools, such as ultrasonic dissecting devices and in-bag morcellation (IBM), allow women with large fibroids or uteri to undergo safe procedures [1]. The use of this approach is an acquired skill for those only familiar with minimally invasive procedures, which avoids the potential inoculation of cells within the abdominal cavity during tissue excision or retrieval [25].

After laparoscopic dissection, the surgical specimen (myomata or uteri) could be retrieved from the abdominal cavity through minilaparotomy through the vagina or by means of morcellation (uncontained or in-bag). The latter refers to reducing the surgical specimen into small fragments with a scalpel (manual) or with an electronic power morcellator (mechanical). In 1973, Kurt Semm introduced the electromechanical power morcellation technique, which was approved by the FDA in 1995 [1]. After 2014, we rarely used manual morcellation. When applying morcellation without a bag, small amounts of tissue dissemination within the abdominopelvic cavity occurs, potentially inoculating myomata cells, which in turn increases the risk of parasitic myoma, endometriosis, and sarcoma development [3]. This occurs because the tissue mass is rotated along with the blade outside the morcellation tube, causing the tissue to spread into the abdomen.

Nevertheless, uterine sarcomas are rare, and the incidence rate differs across countries, being 0.36/100,000 women in the United States, 0.4/100,000 women across North European countries, and 1.32/100,000 women in Germany [3,15,26]. Rapid uterine growth, that is, an increase in size resembling 6 weeks of pregnancy over a period of 1 year, has been accepted as a clinical sign of sarcoma. However, Parker et al. [27] reported that none of the 198 patients (0% incidence) who met a published definition of “rapid growth” had a uterine sarcoma. They also reported an incidence of 0.23% for unexpected malignancy among 1332 patients that underwent leiomyoma surgery, including leiomyosarcoma, endometrial stromal sarcoma, and mixed mesodermal tumor. Only one patient (0.27%), operated on for “rapidly growth” of the uterus, was diagnosed with sarcoma. In addition, the review performed by the DGGG and OEGGG in 2019 did not find a consensus that permits the use of this parameter to differentiate between benign and malignant fibroids [28].

Following myoma surgery, a very low rate of unexpected malignancy has been reported. In 2013, Theben et al. found an unexpected malignancy rate (leiomyosarcoma or endometrial cancer) of 0.25% in 1584 patients who underwent LASH [29]. In 2014, the FDA estimated the risk of uterine sarcoma as 1:350 women undergoing hysterectomy or myomectomy because of myomata [FDA 2014]. In the same year, the AAGL reported that 1 in 400 to 1 in 1000 morcellated, presumed benign specimens are leiomyosarcoma [3]. In 2015, Bojahr et al. reported a very small incidence of sarcoma (0.06%) and endometrial carcinoma (0.07%) in 10,731 patients who underwent standardized LASH surgery [15]. In our series, two cases of sarcoma (0.18%) were suspected intraoperatively. Both were premenopausal women that exhibited tissue fragility at grasping. One case was a 259 g myoma, and the other a 3183 g uteri. Peritoneal washing was negative in both cases. Although in our study, most of the women presented with large myomata, our incidence was between the ranges of the aforementioned studies.

Regarding sarcoma prognosis, tumor injury during excision and uncontained morcellation, electric or manual, in both laparotomy and laparoscopy [30,31,32], plays a critical role in upgrading metastasis, resulting in poorer prognosis [33,34,35,36,37]. Hence, several prognostic factors for all histological uterine sarcoma types have been identified, including patient age, tumor stage, mitotic index, vascular invasion, and tumor-free resection margins. The stage is the most important factor, given that the 5-year overall survival rate is from 50 to 55% for stage I and 8 to 12% for stage II–IV disease. The recurrence rate ranges from 53 to 71% [15,37]. Low-grade stromal sarcoma has a better prognosis, with an overall 5-year survival rate of between 100% and 40% for early and advanced stages, respectively [37].

Further studies have shown that the risk of benign diseases following laparoscopic morcellation is higher than for sarcoma. Tulandi et al. [38] reviewed 51 studies and reported that uncontained morcellation is associated with a risk of iatrogenic endometriosis (1.4%), adenomyosis (0.57%), parasitic myoma (0.9%), and disseminated peritoneal leiomyomatosis (<0.01). Van der Meulen et al. [39] reported an overall incidence of parasitic myomata of 0.12–0.95% in 69 cases (mean age 40.8 ± 7.5 years; range 24–57) from 44 studies reviewed, with a median time between surgery and diagnosis of 48.0 months (range 1–192) and a mean number of parasitic myomas of 2.9 ± 3.3 (range 1–16). Parasitic leiomyomatosis produces late symptoms, which is why it is diagnosed several years after its inoculation or when a tumor-related complication appears [40,41,42,43]. In our series, 10 patients presented with parasitic myomata; all of them had had previous myoma surgery. None of our cases presented parasitic myoma during the 3-year follow-up after IBM.

The concern for occult cancer risk and parasitic myomata may compromise the benefits of minimal-access surgery for uterine leiomyoma. However, the standard use of containment systems, as advised by the FDA in 2020, could help to reduce the risk of dissemination by electromechanical morcellation. Recent studies have shown high technical success and a short learning curve in the technique of in-bag morcellation [1,8,18,43,44,45,46,47]. Our results show that this method is feasible even for the manipulation of large surgical specimens in patients who have undergone an appropriate preoperative evaluation. The largest specimen of multiple myomata weighed 4780 g, and a single uterus weighed 3183 g. Irrespective of the size of the bags, we did not have any case of bag handling failure or any case of failed morcellation.

Our findings are similar to other studies showing that contained morcellation is a feasible and safe method to retrieve large benign surgical specimens and for suspected cases of cancer. There were no intraoperative complications or postsurgical hernias at the port sites [21,43,47,48,49]. We found that giant tumors (>1000 g) required greater skills for manipulation, thus reducing the possibility of cell spreading without significantly increasing the surgical time when compared with tumors less than 500 g. Two cases of puncture occurred; one of them was noticed immediately when the morcellator blade scraped one edge of the bag and only required the bag to be repositioned. The other case was inadvertent but did not lead to a loss of gas from the bag; it was detected postoperatively at the insufflation and immersion test. The presence of bubbles during postoperative in-water immersion of the bag proves that even inadvertent microruptures occur during morcellation and is part of the IBM standard procedure. In all cases, cytological analysis of peritoneal washings was routinely performed, and there was no evidence of microscopic spillage. In contrast to our findings, Vargas et al. [50] reported that IBM prolonged the surgeries by 26 min (mean: 119.0 ± 55.91 vs. 93.13 ± 44.90; *p* = 0.02), which did not vary significantly by the surgeon. There were no differences regarding specimen weight, complication rate, estimated blood loss, or hospital length of stay between the groups (85 cases vs. 49 controls).

Additionally, the analysis of 252 cases of IBM during total laparoscopic hysterectomies performed by Gil-Gimeno et al. [51] reported a 6% failure rate, mainly due to the inability to insert the specimen into the bag or apparent perforation. Mean bag deployment and extraction times were estimated to be 17 ± 9 and 4 ± 3 min, respectively. The total operative time was 40 min longer for the IBM group than for the uncontained group (170 ± 48 vs. 130 ± 43 min; *p* < 0.001), which was attributed to the higher mean uterine size (580 ± 309 vs. 391 ± 122 g; *p* = 0.01). According to our experience, in minimally invasive centers, the learning curve to acquire skills in the bag unwrapping technique, as well as the manipulation and morcellation of large tumors, is short, helping to reduce the excess time related to this procedure.

In summary, the IBM procedure for large and giant myomas proved to be safe and feasible following standard operating procedures for containment systems. The use of the bag added value to the known advantages of laparoscopic myomectomy, which has been associated with fewer complications than open myomectomy in different RCTs and meta-analyses (pooled OR 0.47, 95% CI 0.26–0.85) [52]. However, worldwide, up to 60% of hysterectomies are still performed by laparotomy, either due to a lack of experience with MIS methods or a lack of availability of the necessary medical equipment to perform endoscopic surgeries [53,54]. This exposes patients to the disadvantages of laparotomies in general, such as increased pain, increased wound infection, and longer hospital stays [55]. A further advantage of closed systems is that intra-abdominal peritoneal lavage is not mandatory once the unperforated pouch has been removed, thus shortening the operation time [1,56,57]. Another aspect to consider is the risk of adhesion formation after myomectomy, which is one of the most adhesiogenic gynecological surgeries. Peritoneal adhesions develop in 23% to 88% of open myomectomies and 15.6% to 22% after laparoscopic approaches, with the lowest incidences seen when antiadhesion agents are used [58]. Obviously, the decision of the surgical approach for myomectomy must be individualized, depending on the clinical conditions of the patient, the number, size, and location of the myomas, the availability of technical resources, as well as the experience of the surgeon.

The strength of this study, the largest ever, is the large weight of the extirpated and morcellated specimens. Similar to other studies, this study has some limitations because of the retrospective setup. Nevertheless, these results add to international knowledge of the clinical outcomes after laparoscopic myoma surgery. Future studies should address the incidence of adhesions related to the use of surgical bags.

## 5. Conclusions

This study is the largest published until now regarding the role of in-bag morcellation in large and giant myomata surgery. As reported in our first large series on IBM [1], the present analysis showed that this approach is a viable method for removing surgical specimens from the abdominal cavity, regardless of their size. This technique did not result in the spread of debris during myoma surgery, eliminating the risk of abdominal tissue dissemination. Bag manipulation took only a short time, and perforations rarely occurred, which were easy to detect intraoperatively.

Appropriate bag size selection and the standardization of IBM surgical techniques save operation time, reduce the risk of bag perforation, and maintain the advantages of MIS. A postmorcellation bag insufflation test is useful to confirm a spill-free extirpation. We recommend performing peritoneal washing when bag perforation is detected. Therefore, adequate training in this technique is required to provide the best possible care for patients. Granted, IBM could be proposed as a standard strategy to reduce the risk of occult sarcoma and parasitic myomata while maintaining the significant advantages of minimally invasive approaches.

## Figures and Tables

**Table 1:** Characteristics of MorSafe® thermoplastic polyurethane endobags.

Bag Size	Bigger Opening Diameter	Telescopic Diameter Required	Volume	Bag Schema
Small (S)	12.5 cm	6 mm	1600	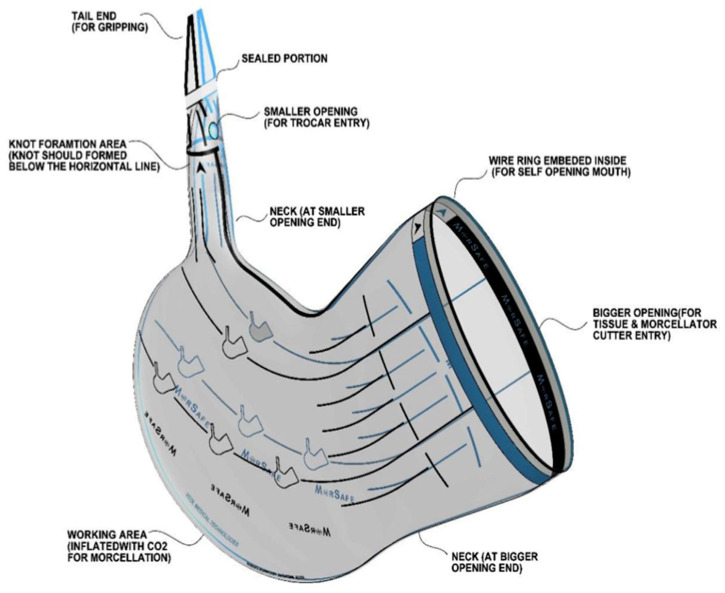
Medium (M)	13.5 cm	6 mm	2100	
Large (L)	14.5 cm	6 mm	2600	
Customized XL variant *	28.5 cm	6 mm	5200/5500	

* Not yet commercially available. MorSafe®, Veol medical technologies PVT LTD, Mumbai, India.

**Table 2:** Patient characteristics, types of surgeries performed, and surgical specimen weight.

Characteristic		Mean (n = 1120)	Range
Patient age (years)		39.3	21–71
Patient weight (kg)		67.0	41–127
Nulliparous		562	NA
Parous		558	1–15
**Type of Surgery**		**n** **= 1120**	**%**
Giant myomectomy		3	0.27
Large myomectomy		744	66.43
Small myomectomy		35	3.12
Parasitic myomectomy		10	0.89
Adenomyomectomy		12	1.07
Giant LASH		1	0.09
Large LASH		148	13.21
Small LASH		67	5.98
Large LASH + BSO		26	2.32
LTH		73	6.52
Retroperitoneal angioleiomyomatosis		1	0.09
**Specimen weight in grams**	**Mean**	**n** **= 1120**	**%**
<250	131.8 ± 0.8	239	21.34%
250–499	349.8 ± 0.8	414	36.96%
500–999	606.4 ± 0.4	386	34.46%
>1000	1718.9 ± 0.9	81	7.23%

LASH, laparoscopic-assisted supracervical hysterectomy; BSO, bilateral salpingo-oophorectomy; LTH, total laparoscopic hysterectomy; NA, not applicable.

**Table 3:** Histological diagnosis.

Type of Tumor	N = 1120	%
Adenomyosis	159	14
Adenomyosis with leiomyoma	14	1
Degenerated myoma	108	10
Leiomyoma	728	65
Leiomyoma cellular variant	28	3
Endometrial complex hyperplasia, polyps	48	4
Endometriosis	26	2
Leiomyosarcoma	2	0.18
Endometrial stromal tumor	1	0.09
Angioleiomyomatosis	1	0.09
No obvious histopathology	5	0.45

**Table 4:** Cytological findings of peritoneal washing samples after in-bag morcellation.

Cytological Description	N = 1120	%
Severe subacute inflammation with activated mesothelial cells and reactive mesothelial cell hyperplasia suggestive of exudative peritoneal effusion	167	15
Mild subacute inflammation with activated mesothelial cells and reactive mesothelial cell hyperplasia	138	12
Cytology of peritoneal fluid with moderate subacute inflammation	136	12
Moderate subacute inflammation consistent with exudative peritoneal effusion	98	9
Severe subacute inflammation with activated hyperplastic mesothelial cells suggestive of exudative peritoneal effusion created by endometriosis	86	8
Mild subacute inflammation suggestive of exudative peritoneal effusion	82	7
Moderate subacute inflammation with circumscribed mesothelial cell hyperplasia suggestive of exudative peritoneal effusion	77	7
Severe subacute inflammation with reactive mesothelial cell hyperplasia suggestive of exudative peritoneal effusion	73	7
Severe acute organizing inflammation with reactive mesothelial cell hyperplasia suggestive of exudative peritoneal effusion	51	5
Severe subacute inflammation with activated mesothelial cells and reactive mesothelial cell hyperplasia suggestive of exudative peritoneal effusion	41	4
Mild to moderate subacute inflammation with reactive mesothelial cell hyperplasia suggestive of exudative peritoneal effusion	39	3
Severe subacute inflammation with mesothelial cell hyperplasia suggestive of exudative peritoneal effusion	25	2
Smear slides of peritoneal fluid showing clear background with scattered mononucleated and mesothelial cells	7	1
Components of the previously diagnosed simple serous papillary cystadenoma of the right ovary	1	0.09
Severe subacute inflammation suggestive of exudative peritoneal effusion. Activated mesothelial cells and abundant mesothelial cell hyperplasia associated with papillary structures. Clinical correlation ruled out intra-abdominal serous papillary neoplasm	1	0.09

**Table 5:** Surgical bag manipulation time.

	Mean Specimen Weight (g)	Bag Manipulation Average Time H:M:S	Morcellation Average Time H:M:S
Time	701.7	0:17:38	0:31:25
Range	8–4780	0:0:30–0:48:32	0:1:16–1:31:50

## Data Availability

Anonymized data set is available upon request from the Department of Obstetrics and Gynecology, Minimal-Access Surgery and Oncology, Dubai London Clinic and Speciality Hospital, Dubai, UAE.

## References

[1] Devassy R., Cezar C., Krentel H., Verhoeven H.C., Devassy R., de Wilde M.S., Torres-de la Roche L.A., Wilde R.L. (2019). Feasibility of myomatous tissue extraction in laparoscopic surgery by contained in—Bag morcellation: A retrospective single arm study. Int. J. Surg..

[2] Cezar C., Korell M., Tchartchian G., Ziegler N., Senshu K., Herrmann A., Larbig A., De Wilde R.L. (2015). How to avoid risks for patients in minimal-access trials: Avoiding complications in clinical first-in-human studies by example of the ADBEE study. Best Pract. Res. Clin. Obstet. Gynaecol..

[3] Brown J. (2014). AAGL Advancing Minimally Invasive Gynecology Worldwide: Statement to the FDA on Power Morcellation. J. Minim. Invasive Gynecol..

[4] Herrmann A., De Wilde R.L. (2014). Laparoscopic myomectomy—The gold standard. Gynecol. Minim. Invasive Ther..

[5] AAGL Advancing Minimally Invasive Gynecology Worldwide (2011). AAGL Position Statement: Route of Hysterectomy to Treat Benign Uterine Disease. J. Minim. Invasive Gynecol..

[6] US Food and Drug Administration Laparoscopic Uterine Power Morcellation in Hysterectomy and Myomectomy: FDA Safety Communication. http://www.fda.gov/MedicalDevices/Safety/AlertsandNotices/ucm393576.htm.

[7] US Food and Drug Administration Safety Communication Perform Only Contained Morcellation When Laparoscopic Power Morcellation Is Appropriate. 29 December 2020 Update. https://www.fda.gov/medical-devices/safety-communications/update-fda-recommends-performing-contained-morcellation-women-when-laparoscopic-power-morcellation.

[8] Cohen S.L., Einarsson J.I., Wang K.C., Brown D., Boruta D., Scheib S.A., Fader A.N., Shibley T. (2014). Contained Power Morcellation within an Insufflated Isolation Bag. Obstet. Gynecol..

[9] Srouji S.S., Kaser D.J., Gargiulo A.R. (2015). Techniques for contained morcellation in gynecologic surgery. Fertil. Steril..

[10] Semm K. (1991). Morzellieren und Nähen per pelviskopiam—Kein Problem mehr. Geburtshilfe Frauenheilkd..

[11] Leren V., Langebrekke A., Qvigstad E. (2012). Parasitic leiomyomas after laparoscopic surgery with morcellation. Acta Obstet. Gynecol. Scand..

[12] Bortoletto P., Friedman J., Milad M.P. (2018). Economics of gynecologic morcellation. Curr. Opin. Obstet. Gynecol..

[13] Cusidó M., Fargas F., Baulies S., Plana A., Rodríguez I., Tresserra F., Pascual M., Fábregas R. (2015). Impact of Surgery on the Evolution of Uterine Sarcomas. J. Minim. Invasive Gynecol..

[14] Kho K.A., Anderson T.L., Nezhat C.H. (2014). Intracorporeal Electromechanical Tissue Morcellation: A critical review and recommen-dations for clinical practice. Obstet. Gynecol..

[15] Bojahr B., De Wilde R.L., Tchartchian G. (2015). Malignancy rate of 10,731 uteri morcellated during laparoscopic supracervical hysterectomy (LASH). Arch. Gynecol. Obstet..

[16] Brölmann H., Tanos V., Grimbizis G., Ind T., Philips K., Bosch T.V.D., Sawalhe S., Haak L.V.D., Jansen F.-W., Pijnenborg J. (2015). Options on fibroid morcellation: A literature review. Gynecol. Surg..

[17] Milad M.P., Milad E.A. (2014). Laparoscopic Morcellator-Related Complications. J. Minim. Invasive Gynecol..

[18] Cholkeri-Singh A., Miller C.E. (2015). Power Morcellation in a Specimen Bag. J. Minim. Invasive Gynecol..

[19] Zaami S., Zupi E., Lazzeri L., Stark M., Malvasi A., Signore F., Marinelli E. (2020). Medicolegal Issues in Power Morcellation: Cautionary Rules for Gynecologists to Avoid Unfavorable Outcomes. J. Minim. Invasive Gynecol..

[20] Xu X., Desai V.B., Wright J.D., Lin H., Schwartz P.E., Gross C.P. (2020). Hospital variation in responses to safety warnings about power morcellation in hysterectomy. Am. J. Obstet. Gynecol..

[21] Torres-de la Roche L.A., Devassy R., Makhlouf G., Juan J.S., Eidswick J., De Wilde R.L. (2020). Retroperitoneal angioleiomyomatosis. J. Obstet. Gynecol. India.

[22] Torres-de la Roche L.A., Becker S., Cezar C., Hermann A., Larbig A., Leicher L., Sardo A.D.S., Tanos V., Wallwiener M., Verhoeven H. (2017). Pathobiology of myomatosis uteri: The underlying knowledge to support our clinical practice. Arch. Gynecol. Obstet..

[23] Shifrin G., Engelhardt M., Gee P., Pschadka G. (2021). Transcervical fibroid ablation with the Sonata™ system for treatment of submucous and large uterine fibroids. Int. J. Gynecol. Obstet..

[24] Gothwal M., Devkare V. (2021). Successful laparoscopic myomectomy in giant myoma. Int. J. Appl. Basic Med. Res..

[25] AAGL Advancing Minimally Invasive Gynecology Worldwide (2014). AAGL Practice Report: Morcellation during Uterine Tissue Extraction. J. Minim. Invasive Gynecol..

[26] Koivisto-Korander R., Martinsen J., Weiderpass E., Leminen A., Pukkala E. (2012). Incidence of uterine leiomyosarcoma and endometrial stromal sarcoma in Nordic countries: Results from NORDCAN and NOCCA databases. Maturitas.

[27] Parker W.H., Fu Y.S., Berek J.S. (1994). Uterine sarcoma in patients operated on for presumed leiomyoma and rapidly growing leio-myoma. Obstet. Gynecol..

[28] Denschlag D., Ackermann S., Battista M.J., Cremer W., Egerer G., Follmann M., Haas H., Harter P., Hettmer S., Horn L.-C. (2019). Sarcoma of the Uterus. Guideline of the DGGG and OEGGG (S2k Level, AWMF Register Number 015/074, February 2019). Geburtshilfe Frauenheilkd..

[29] Theben J.U., Schellong A.R.M., Altgassen C., Kelling K., Schneider S., Große-Drieling D. (2012). Unexpected malignancies after laparoscopic-assisted supracervical hysterectomies (LASH): An analysis of 1584 LASH cases. Arch. Gynecol. Obstet..

[30] Seidman M.A., Oduyebo T., Muto M.G., Crum C.P., Nucci M.R., Quade B.J. (2012). Peritoneal Dissemination Complicating Morcellation of Uterine Mesenchymal Neoplasms. PLoS ONE.

[31] Grady D. (2016). Uterine Surgical Technique Is Linked to Abnormal Growths and Cancer Spread. The New York Times. http://www.nytimes.com/2014/02/07/health/uterine-surgical-technique-is-linked-totoabnormal.

[32] Park J.-Y., Park S.-K., Kim D.-Y., Kim J.-H., Kim Y.-M., Kim Y.-T., Nam J.-H. (2011). The impact of tumor morcellation during surgery on the prognosis of patients with apparently early uterine leiomyosarcoma. Gynecol. Oncol..

[33] De Wilde R.L. (2017). Regarding “Incidence of Occult Uterine Malignancy Following Vaginal Hysterectomy with Morcellation”. J. Minim. Invasive Gynecol..

[34] Graebe K., Garcia-Soto A., Aziz M., Valarezo V., Heller P.B., Tchabo N., Tobias D.H., Salamon C., Ramieri J., Dise C. (2015). Incidental power morcellation of malignancy: A retrospective cohort study. Gynecol. Oncol..

[35] Wright J.D., Tergas A.I., Cui R., Burke W.M., Hou J.Y., Ananth C.V., Chen L., Richards C., Neugut A.I., Hershman D.L. (2015). Use of Electric Power Morcellation and Prevalence of Underlying Cancer in Women Who Undergo Myomectomy. JAMA Oncol..

[36] Turner T., Secord A.A., Lowery W.J., Sfakianos G., Lee P.S. (2013). Metastatic adenocarcinoma after laparoscopic supracervical hysterectomy with morcellation: A case report. Gynecol. Oncol. Case Rep..

[37] Huss A., Klar M., Hasanov M.F., Juhasz-Böss I., Bossart M. (2022). Prognostic factors and survival of patients with uterine sarcoma: A German unicenter analysis. Arch. Gynecol. Obstet..

[38] Tulandi T., Leung A., Jan N. (2016). Nonmalignant Sequelae of Unconfined Morcellation at Laparoscopic Hysterectomy or Myomectomy. J. Minim. Invasive Gynecol..

[39] Van Der Meulen J.F., Pijnenborg J., Boomsma C.M., Verberg M.F.G., Geomini P.M.A.J., Bongers M.Y. (2015). Parasitic myoma after laparoscopic morcellation: A systematic review of the literature. BJOG Int. J. Obstet. Gynaecol..

[40] Mowers E.L., Skinner B., McLean K., Reynolds R.K. (2015). Effects of Morcellation of Uterine Smooth Muscle Tumor of Uncertain Malignant Potential and Endometrial Stromal Sarcoma: Case Series and Recommendations for Clinical Practice. J. Minim. Invasive Gynecol..

[41] Cucinella G., Granese R., Calagna G., Somigliana E., Perino A. (2011). Parasitic myomas after laparoscopic surgery: An emerging complication in the use of morcellator? Description of four cases. Fertil. Steril..

[42] Panesar H., Dhaliwal H.S. (2022). Iatrogenic parasitic leiomyomas: A late and uncommon complication after laparoscopic morcellation. Cureus.

[43] Wong M., De Wilde R.L., Isaacson K. (2017). Reducing the spread of occult uterine sarcoma at the time of minimally invasive gynecologic surgery. Arch. Gynecol. Obstet..

[44] Krentel H., De Wilde R.L. (2017). Laparoscopic Supracervical Hysterectomy with In-Bag Morcellation in Very Large Uterus. Case Rep. Med..

[45] Rimbach S., Holzknecht A., Schmedler C., Nemes C., Offner F. (2015). First clinical experiences using a new in-bag morcellation system during laparoscopic hysterectomy. Arch. Gynecol. Obstet..

[46] Rimbach S., Schempershofe M. (2017). In-Bag Morcellation as a Routine for Laparoscopic Hysterectomy. BioMed Res. Int..

[47] Rimbach S., Holzknecht A., Nemes C., Offner F., Craina M. (2015). A new in-bag system to reduce the risk of tissue morcellation: Development and experimental evaluation during laparoscopic hysterectomy. Arch. Gynecol. Obstet..

[48] Kanade T.T., McKenna J.B., Choi S., Tsai B.P., Rosen D.M., Cario G.M., Chou D. (2014). Sydney Contained in Bag Morcellation for Laparoscopic Myomectomy. J. Minim. Invasive Gynecol..

[49] Serur E., Zambrano N., Brown K., Clemetson E., Lakhi N. (2016). Extracorporeal Manual Morcellation of Very Large Uteri within an Enclosed Endoscopic Bag: Our 5-Year Experience. J. Minim. Invasive Gynecol..

[50] Vargas M.V., Cohen S.L., Fuchs-Weizman N., Wang K.C., Manoucheri E., Vitonis A.F., Einarsson J.I. (2015). Open Power Morcellation Versus Contained Power Morcellation Within an Insufflated Isolation Bag: Comparison of Perioperative Outcomes. J. Minim. Invasive Gynecol..

[51] Gil-Gimeno A., Laberge P.Y., Lemyre M., Gorak E., Maheux-Lacroix S. (2020). Morcellation During Total Laparoscopic Hysterectomies: Implications of the Use of a Contained Bag System. J. Obstet. Gynaecol. Can..

[52] Jin C., Hu Y., Chen X.-C., Zheng F.-Y., Lin F., Zhou K., Chen F.-D., Gu H.-Z. (2009). Laparoscopic versus open myomectomy—A meta-analysis of randomized controlled trials. Eur. J. Obstet. Gynecol. Reprod. Biol..

[53] Donnez O., Jadoul P., Squifflet J., Donnez J. (2008). A series of 3190 laparoscopic hysterectomies for benign disease from 1990 to 2006: Evaluation of complications compared with vaginal and abdominal procedures. BJOG Int. J. Obstet. Gynaecol..

[54] Wright J.D., Herzog T.J., Tsui J., Ananth C.V., Lewin S.N., Lu Y.-S., Neugut A.I., Hershman D.L. (2013). Nationwide Trends in the Performance of Inpatient Hysterectomy in the United States. Obstet. Gynecol..

[55] Cezar C., Becker S., di Spiezio Sardo A., Herrmann A., Larbig A., Tanos V., Torres-de la Roche L.A., Verhoeven H.C., Wallwiener M., De Wilde R.L. (2017). Laparoscopy or laparotomy as the way of entrance in myoma enucleation. Arch. Gynecol. Obstet..

[56] Nieboer T., Johnson N., Lethaby A., Tavender E., Curr E., Garry R., Van Voorst S., Mol B.W.J., Kluivers K.B. (2009). Surgical approach to hysterectomy for benign gynaecological disease. Cochrane Database Syst. Rev..

[57] Taylan E., Sahin C., Zeybek B., Akdemir A. (2017). Contained Morcellation: Review of Current Methods and Future Directions. Front. Surg..

[58] De Wilde R.L., Herrmann A., Torres-de la Roche L.A., Krentel H., Cezar C., de Wilde M.S., Devassy R. (2020). Adhesions after laparoscopic myomectomy: Incidence, risk factors, complications, and prevention. Gynecol. Minim. Invasive Ther..

